# Early complementary acupuncture improves the clinical prognosis of traumatic brain edema: A randomized controlled trial

**DOI:** 10.1097/MD.0000000000028959

**Published:** 2022-02-25

**Authors:** Zi-Quan Guo, Hua Jiang, Yong Huang, Hong-Mei Gu, Wen-Bin Wang, Tai-Dong Chen

**Affiliations:** a School of Traditional Chinese Medicine, Southern Medical University, Guangzhou, China; b Neurosurgery Center of Qionghai People's Hospital, Qionghai, China; c Department of Acupuncture of Qionghai People's Hospital, Qionghai, China; d Nanfang Hospital, Southern Medical University, Guangzhou, China; e School of Public Health, Mudanjiang Medical College, Mudanjiang, China.

**Keywords:** acupuncture, acute-phase traumatic brain injury, long-term effects, randomized controlled trial, traumatic brain edema

## Abstract

**Background:**

Traumatic brain edema occurs commonly brain injury, and most manifests as pericontusional edema of brain contusions. On the basis of evidence-based medicine, apart from recommending craniotomy and mannitol, there are few particularly effective measures to prevent and treat traumatic brain edema. It is uncertain whether an early complementary acupuncture treatment would improve long-term outcomes of patients with traumatic brain edema. The aim of this study is to assess the efficacy and the safety of early complementary acupuncture for patients with traumatic brain edema.

**Methods:**

This study is an actively accruing, single-center, single-blinded, 2-arm, randomized controlled trial. Patients with traumatic brain injury, a Glasgow Coma Scale score of 6∼12, and brain edema on computed tomography scan will be divided into 2 groups on the basis of stratified block randomization. All patients will receive conventional treatment, and the study group will undergo additional acupuncture therapy (start within 72 hours after the injury) once a day for 28 days. The primary outcome is the dichotomized Glasgow Outcome Score at 6 months and 12 months after injury, and the secondary outcomes are the Glasgow Coma Scale, the volume of traumatic brain edema, the serum levels of C-reactive protein and interleukin-6, and the Modified Barthel Index.

**Discussion:**

This study will provide data regarding the efficacy of early complementary acupuncture for traumatic brain edema. If the study yields positive results, its findings may offer insights into a valuable complementary option of acupuncture for traumatic brain edema that could provide pilot evidence for large, randomized, controlled trials.

**Trial registration:** This trial has been published in the Chinese Clinical Trial Register, http://www.chictr.org.cn/edit.aspx?pid=141208&htm=4 (Identifier: ChiCTR2100053794, registered on December 3, 2021).

## 1 Introduction

Traumatic brain edema (TBE) is defined as a pathological condition in which the fluid content in the brain tissue increases abnormally and the brain volume increases after traumatic brain injury (TBI) or craniotomy; TBE commonly manifests as pericontusional edema after brain contusions.^[1]^ Localized cerebral edema, erupting simultaneously with brain contusions, might lead to increased intracranial pressure (ICP), cerebral herniation, and ultimately death.^[2]^ TBE, an independent prognostic variable of all severe TBI, is the most common and serious complication of TBI. TBE contributes to approximately 50% of the deaths of patients with severe TBI.^[3]^ In China, 53.17% of patients experience unfavorable outcomes from TBI, which include severe disability, a vegetative state, and death.^[4]^ As medicine has advanced rapidly, the survival of patients with TBI has also improved in recent years. However, the long-term treatments needed for the survivor place inconveniences and financial pressures on the family and society.^[5]^

TBE is a major cause of death and severe, prolonged disability in patients with TBI. Research on the treatment and the prevention of TBE has continued for more than half a century, but there has been no breakthrough in this field. Evidence from evidence-based medicine have shown that only few other treatments have definite efficacy for TBE beyond craniotomy and mannitol.^[6]^ However, craniotomy and mannitol treatment have limitations. For example, brain edema may progress after craniotomy, and mannitol may cause adverse effects that include ICP rebound and kidney damage.^[7]^

Although effective therapeutic approaches for TBE remain limited, researchers continue to make meaningful attempts to determine effective and alternative treatment options, such as acupuncture for TBE. For patients who are unconscious after TBI, acupuncture may improve the Glasgow Coma Scale (GCS) score^[8,9]^; increase wake-promoting rates (Wake-promoting rates refers to the percentage of patients who recovered to GCS 15 to the total number of patients in this group at the end of treatment.)^[10,11]^; and change the GOS grade,^[12]^ which improves the prognosis. However, studies conducted previously by our team have demonstrated the fact that acupuncture could promote the absorption of traumatic intracerebral hematoma and brain contusion lesions, and improve the quality of life of such patients.^[13,14]^ Even though the acupuncture has been observed to be an effective treatment for patients with TBI, some of the acupuncture data remain weak and unconvincing.^[15]^ Few studies have investigated acupuncture as an adjuvant therapy in the acute phase of TBI (within 2 weeks after injury), which high-quality randomized controlled trials (RCTs) are particularly lacking.^[16]^ For this reason, and taking into account the preliminary results of the previous trials, a standardized RCT is planned to assess the effect of acupuncture on the prognosis of patients with TBE. The method of monitoring and quantitatively analyzing brain edema based on head computed tomography (CT) can be very convincing, and complementary acupuncture treatment in the acute-phase TBI (starting within 72 hours after injury) can be a novel idea of designing in this clinical trial.

If TBE is not controlled, the ICP will continue to increase, which can lead to fatal brain herniation.^[6]^ This unfavorable prognosis of TBE emphasizes the importance of managing the increased ICP in patients with TBE. Although the technology of ICP monitoring is precise and accurate, its invasive operation possibly harms patients.^[17]^ As a noninvasive examination for TBI, CT scans play a vital role in the study of brain edema; the CT images can evaluate ICP at an acute phase TBI, and software is used to reduce the subjectiveness of quantifying brain edema.^[18,19]^

The pathological mechanism of TBE is unclear. After injury, the neuroinflammatory cascade that is considered an important mechanism of TBE promotes the rapid progression and progressive aggravation of TBE.^[20]^ Highly expressed inflammatory factors in the damaged brain tissue [e.g., C-reactive protein (CRP) and interleukin-6 (IL-6)] are released into the blood circulation; then, the degree of nerve tissue damage and the prognosis can be judged according to the serum levels of inflammatory factors.^[21]^ Many clinical studies have provided evidence of the anti-inflammatory effects of acupuncture. For example, Chen et al^[22]^ have concluded that acupuncture could downregulate the levels of inflammatory factors (IL-6 and IL-8) in patients with TBI, whereas Cho et al^[23]^ have found that acupuncture could reduce CRP levels in patients after craniotomy.

TBE is a complex and changeable pathological process, and the appropriate target cohort of treatment should be selected in order to measure the effectiveness of acupuncture. Here, we present the study protocol for a RCT to examine whether complementary acupuncture on acute-phase TBI is effective compared against the conventional treatment alone in patients with TBE. In addition, the study will also observe whether acupuncture is associated with an anti-inflammatory effect and a reduction in the volume of brain edema.

We present the following article in accordance with the SPIRIT reporting checklist.

## 2 Methods/design

### 2.1 Trial design and setting

This study is a single-center, outcome-assessor, and statistician-blinded, randomized, and controlled trial that was devised according to the Standards for Reporting Interventions in Clinical Trials of Acupuncture.^[24]^ The study consists of 4 stages: enrollment, allocation, intervention, and analysis. Each participant will be registered only once. The total study period for this trial will be 12 months (follow-up to 12 months after injury), including a 1-month treatment phase. This is the initial version of the protocol. The start of this study will been plan on January 10, 2022, and ends on June 10, 2024. It may be completed ahead of schedule if a sufficient number of patients are recruited.

The study will be carried out in the Neurosurgery Center of Qionghai People's Hospital, Hainan Province, China, and all participants will be recruited from the inpatients in this center. A minimum of 40 patients who meet the eligibility criteria and sign an informed consent form are expected in this hospital each year. Patients with TBI (GCS score of 6∼12 and brain edema on CT scan) will be divided into 2 groups; the control group will receive conventional treatment, and the study group will receive additional acupuncture as an adjuvant therapy, once a day for 28 days, starting within 72 hours after the injury.

### 2.2 Recruitment strategies

**Figure 1:**
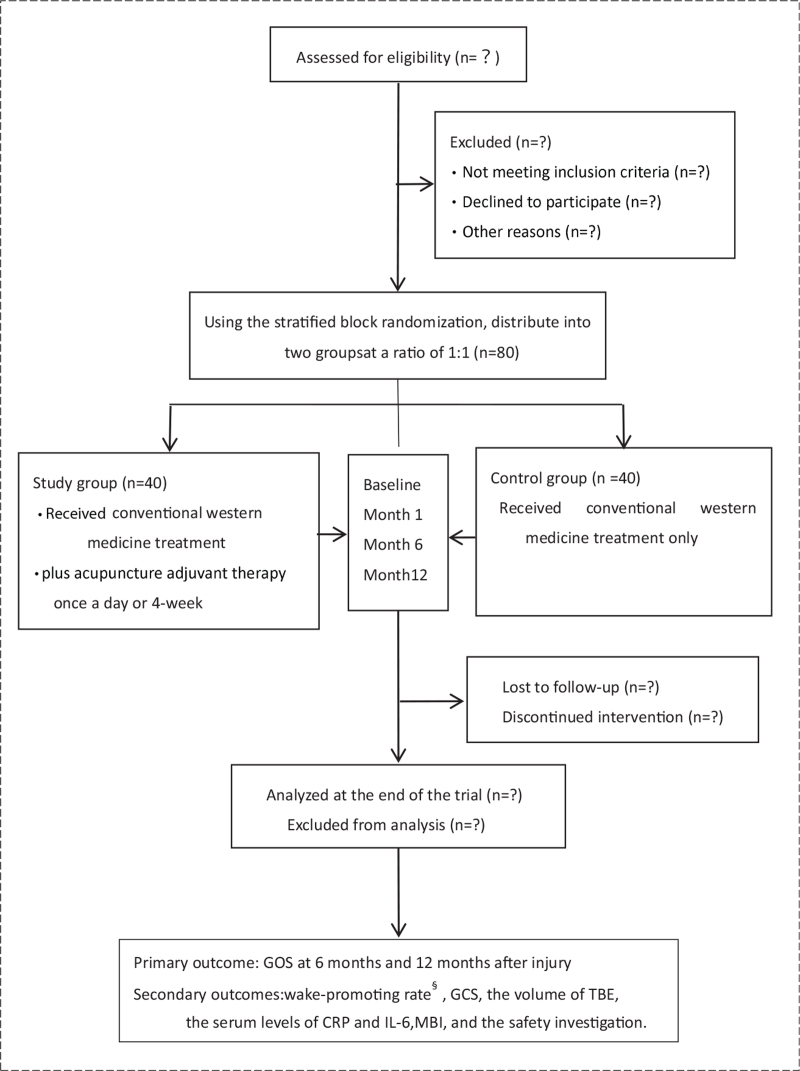
Flowchart of enrollment, allocation, interventions, and analysis in the trial. CRP = C-reactive protein, GCS = Glasgow Coma scale, GOS = Glasgow outcome score, IL-6 = interleukin-6, MBI = Modified Barthel Index, TBE = Traumatic brain edema. ^*^Wake-promoting rates refers to the percentage of patients who recovered to GCS 15 to the total number of patients in this group at the end of 28 days of treatment.

Inpatients at the Neurosurgery Center of Qionghai People's Hospital, Hainan Province, China, will be recruited in this RCT. Patients with a GCS score of 12 or lower would not be capable of giving informed consent to participate in a clinical trial, so all relatives (or legal representatives) of patients will be able to consent to participate or drop out at any time. The responsible doctor will briefly explain to the relatives that the patient will receive the usual treatments for TBE but that, in addition to these, the patient in the study group will also undergo acupuncture as an adjuvant therapy. The benefits and possible risks of the trial will be described, and patient representatives will be required to sign the informed consent before the trial begins. The flowchart of enrollment, allocation, interventions, and analysis is presented in Figure 1.

### 2.3 Participants

#### 2.3.1 Eligibility criteria: inclusion criteria

Adults with TBI who fulfill the following inclusion criteria are eligible: an initial GCS score between 6 and 12 on admission; one or more brain contusions with visible pericontusional edema on the CT scan; age between 16 and 75 years; agreement to undergo acupuncture treatment within 72 hours after injury or craniotomy; and completion of informed consent by a legal representative.

#### 2.3.2 Eligibility criteria: exclusion criteria

The exclusion criteria are as follows: unstable vital signs within 72 hours of admission; a concomitant, severe medical disease (e.g., heart, liver, renal failure, or an autoimmune disorder); craniotomy before random assignment for any reason other than TBI; unsuitability for acupuncture (e.g., pregnant, diagnosed with frequent seizures or psychotic disorders, fear of acupuncture, particularly weak, skin infections at the acupuncture site); and need for medication to treat coexisting diseases that may affect the results of the study.

### 2.4 Randomization and blinding

To ensure a balanced sample size across groups over time, the study is using stratified block randomization in a 1:1 ratio that divides patients into 2 groups according to the GCS (scores of 6∼8 or 9∼12). The study will also apply block randomization method with a block size of 2 (post-craniotomy or no craniotomy).

Random numbers will be made by a computerized number generator list provided by a statistician not involved in the determination of eligibility or in the assessment of outcomes. The allocation of participants will be sealed in sequentially numbered, opaque envelopes.

After verification that a patient meets all the inclusion criteria and none of the exclusion criteria, and after the informed consent is signed by legal representative, the doctor responsible for the patient will assign a correlative number to the patient who will be allocated to either the study group or the control group.

The blinding of this study is limited because it is easily discernible that some patients are receiving acupuncture therapy, and the acupuncturist cannot be blinded. However, the outcome assessors and statistician will be blinded and unaware of patient allocation. During the whole research process, unblinding will be carried out after the statistical analysis is completed.

### 2.5 Interventions

#### 2.5.1 Interventions

In addition to conventional treatment, patients in the study group will receive acupuncture as adjuvant therapy, which will start within 72 hours after injury or craniotomy; acupuncture will be administered once a day for 28days (a total of 24 treatments with 1 break per week). All interventions will be performed by the same acupuncturist doctor, who has more than 10 years of clinical experience and who will be trained in the study protocol before the start of the trial. Encouragement and supervision from acupuncturists can help improve participants’ adherence.

Only sterile, stainless, disposable acupuncture needles (size: from 0.25 × 30 mm to 0.25 × 50 mm; Suzhou Hwato Acupuncture Medical Appliance Company, China; product no. 20162270970) will be used. In the study group, acupuncture needles will be inserted into acupuncture points located on the midline of the human body (GV20, GV26, and CV9) and on the affected limbs (GB20, GB39, PC6, LI4, ST36, ST40, SP6, KI7, and LR3). If the GV20 point is too close to the surgical site, this acupoint may be skipped. All the acupoints have been selected and localized according to the guideline of WHO Standardized Acupuncture Point Location, and then depending on where the needle is located the insertion depth will be 10 to 40 mm.^[25]^ The needles will be manipulated using twirling, lifting, and thrusting methods to promote Qi. Because patients are not able to express their feelings, eye moisture or the presence of tears will be considered as a kind of Deqi. After insertion, needles will be left in position for 30 minutes in every session and the manipulations of twirling, lifting, and thrusting will be repeated for 1 minute/10 minutes. The location of the relevant acupoints is summarized in Table 1.

**Table 1:** Description of the location of the applied acupoints.

Acupoints	Descriptions
Baihui (GV20)	Straight up 5 cun above the middle of the front hairline
Shuigou (GV26)	At the junction of the upper and middle third of the philtrum
Fengchi (GB20)	Below the occiput, the depression between the upper end of the trapezius muscle and the upper end of the sternocleidomastoid muscle.
Xuanzhong (GB39)	On the outside of the calf, 3 cun above the tip of the lateral malleolus, and the depression of the front edge of the fibula
Shuifen (CV9)	On the upper abdomen, anterior midline, 1 cun above the middle of the umbilicus
Neiguan (PC6)	2 cun above the transverse crease of the wrist, between the palmar longus tendon and the flexor carpi radialis tendon
Hegu (LI4)	On the back of the hand, at the midpoint of the radial side of the second metacarpal bone
Zusanli (ST36)	3 cun down of the depression on the lateral side of the knee and 1 cun away of the front edge of the tibia
Fenglong (ST40)	On the anterolateral side of the calf, 8 cun above the tip of the lateral malleolus, the outer edge of the tibial anterior muscle
Sanyinjiao (SP6)	3 cun above the tip of the medial malleolus, posterior tibia
Fuliu (KI7)	2 cun above the tip of the medial malleolus, the front edge of the Achilles tendon
Taichong (LR3)	In the back of the foot, between the first and second metatarsals, in the anterior depression of the metatarsal junction

The “cun” in acupuncture is the bone-length proportion, which is divided by the body surface mark of the patient him or herself.

#### 2.5.2 Conventional treatment in both groups

According to the clinical guidelines for the treatment of TBE, all the participants will undergo conventional treatment, which involves general management in the Department of Neurosurgery to stabilize vital signs, the medicine or the surgery to reduce ICP, neuroprotective medicines, treatment of infections, and other intravenously administered fluid or drug therapy required according to the condition of the patient. However, it is not allowed to give patients treatments that reduce ICP or improve brain function outside of this study. During the clinical trial period, all treatments, including acupuncture needles and medications, will be inspected by investigators who will record any changes and the reasons for them.

#### 2.5.3 Discontinuing interventions

The trial will be ceased if one of the following conditions appears: development of serious complications or any other severe condition that leaves the patient in a critical condition; serious adverse reactions and an inability to continue the trial; withdrawal of consent; or a decision by the principal investigator that it is not possible for the patient to participate in the study as planned.

### 2.6 Outcome measures

#### 2.6.1 Primary outcome

The primary outcome is the functional status of patients with TBE according to a dichotomized GOS (poor recovery = GOS 1∼3; good recovery = GOS 4∼5) at 6 and 12 months after the injury.

#### 2.6.2 Secondary outcomes

The secondary outcomes are wake-promoting rates, changes in the GCS (Baseline GCS is used as the basis for grouping), the volume of TBE calculated by CT scan (i.e., the volume of brain contusions and pericontusional edema), and the serum levels of CRP and IL-6 measured at four different time points (baseline and days 7, 14, and 28 after treatment). Another secondary outcome, the Modified Barthel Index (MBI), will be measured at the beginning of treatment, at the end of 28-day treatment, at the 6-month and 12-month follow-up after injury.

Calculating the volume of TBE, including the volume of brain contusions and pericontusional edema, by CT scan with the help of 3D Slicer software (version 4.8.1) might reduce the subjective impact of quantifying brain edema. 3D Slicer is a free, open source, and multi-platform software package widely used for medical, biomedical, and related imaging research; it has been jointly developed by multiple institutions and has been supported by the National Institutes of Health in the United States.

The Human IL-6 ELISA kit (Wuhan Fine Biotech Co., Ltd., China) or the Human C-Reactive Protein ELISA kit (Head Office in China Beckman Coulter Commercial Enterprise (China) Co., Ltd. Shanghai, China) will be used to determine the concentrations of IL-6 or CRP in the samples, and the data will be analyzed. Collected samples will be centrifuged for 15 minutes at 1000 × *g* and stored in a freezer at −80 C until they are ready for processing and analysis. Samples will be discarded after the analysis. The timepoints for assessment are listed in Table 2.

**Table 2:**
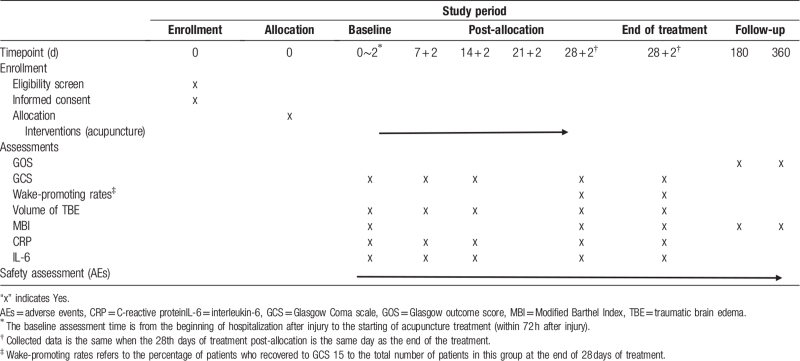
Schedule of enrollment, interventions, and assessment.

#### 2.6.3 Safety assessment

Safety will be assessed by monitoring adverse events. Any adverse events or abnormalities that may occur in this study, including bleeding, local hematoma, infection, pallor, sweating or dizziness, fainting during acupuncture treatment, and objective worsening of existing symptoms, will be voluntarily reported and documented on case report forms (CRFs). The CRFs will record the adverse events in detail, including the time and date of occurrence, degree of severity, any measurement related to the treatment of the event, and any potentially causal relationship between the treatment and the event. If any severe adverse event occurs, acupuncture intervention will be ceased immediately, and the principal investigator will be informed and will take proper actions. It is required to report the events and actions that have occurred to the ethics committee of the hospital.

### 2.7 Follow-up

After 4 weeks of complement acupuncture treatment, the trial will enter a follow-up period till 12 months after injury, when all participants receive the equal therapy as possible follow-up on a monthly basis via telephonic and message communications or outpatient review, treatment compliance and changes of clinical manifestations will be faithfully recorded. The data of 6-month and 12-month will be used for the final statistical analysis of the trial.

### 2.8 Data collection

Participants’ identification records will be kept confidential until the results of the study are published. All the study data will be collected from the patients’ routine medical records by the investigator and registered on the CRFs. The information on CRFs will then be entered into a predesigned, password-protected electronic data set by 2 independent investigators who will be blinded to group allocation. To ensure accuracy, any corrections made to the CRFs must be personally signed and dated by the person responsible, and another researcher will double-check the entered data. Original paper CRFs will remain at the trial site. The data will be used in accordance with local law and ethics committee approval. The original data will be shared through ResMan, which is the Clinical Trial Management Public Platform (http://www.medresman.org.cn/uc/index.aspx).

The Data and Safety Monitoring Board (DSMB) for this study will supervise the entire process. All members of the DSMB are independent of the study sponsor and have no competing interests.

### 2.9 Sample size calculation

Because the data available for patients with TBE who receive early acupuncture treatment are limited and inconclusive, we will perform a single-blind RCT in which the control group will receive conventional treatment and the study group will receive additional acupuncture as an adjuvant therapy. The primary outcome will be dichotomized GOS (poor recovery = GOS 1∼3; good recovery = GOS 4∼5) at 6 and 12 months of follow-up after the injury. According to our previous pilot study, the change of GOS (good recovery rate) in the study group was shown to be 80% (n = 12), and that in control group was 50% (n = 12). Sample size was calculated using the Power Analysis and Sample Size (PASS) software package (version 15, NCSS, Kaysville, UT), with a 2-sided significance level (α = 0.05), a power of 80%, and chi-squared tests to assess the difference in rates between the 2 groups. Thus, a total sample size of 80 participants (40 per group) will be recruited, allowing for 10% loss during follow-up.

### 2.10 Statistical analysis

After a patient has been randomly assigned, the outcome measures will be collected even if the trial treatment is interrupted or not given. The data used will include the method of the last observation carried forward or the method of the worst observation carried forward.^[26]^ That is, this study will analyze the data with the full analysis set.

The statistical analyses will be performed by a blinded researcher. According to the intention-to-treat principle, all the data will be analyzed using the SPSS statistical software package (version 20.0, IBM SPSS Statistics, IBM Corp, Somers, NY). Baseline characteristics will be described and compared. Continuous variables subject to normal distribution will be presented as the mean ± standard deviation; continuous variables not subject to normal distribution will be expressed as the median and interquartile range; and categorical variables will be described as the frequency and percentage.

GOS, wake-promoting rates, and adverse events rates will be compared using the chi-squared or Fisher's exact tests. GCS and MBI scores will be compared between the 2 groups by *t* test with a 95% confidence interval. The results of the volume of TBE and the serum levels of CRP and IL-6 between the 2 groups will be compared using either a generalized linear mixed model or the *t* test, and repeated contrast tests will be conducted to account for time differences in each group. If the data are not normally distributed, nonparametric methods will be used. For all analyses, the results with a *P* value less than .05 will be considered statistically significant. The clinical efficacy should be determined in the evaluation of clinical significance.

### 2.11 Quality control

The study protocol has been reviewed and revised by experts on neurosurgery, acupuncture, rehabilitation, and statistics several times. Before the trial, all the researchers will be trained to ensure that the personnel involved fully understand the trial protocol and standard operating procedures. All the study data will be recorded on the CRFs. Data will be uploaded and verified by 2 additional researchers who will be unaware of patient allocation. Monitoring and auditing of the clinical trial will be conducted by the DSMB who will verify all documents related to the clinical trial, including CRFs, and monitor whether the clinical trial is conducted in accordance with the prescribed protocols. If any revisions of protocol occur, they must be approved by the ethics committee of the hospital.

### 2.12 Ethics and dissemination

The study protocol conforms to the ethical guidelines of the Declaration of Helsinki^[27]^ and is approved by the Ethics Committee of Qionghai People's Hospital, Hainan Province, China (clinical trial plan approval number: QH065). The trial has been published in the Chinese Clinical Trial Register, http://www.chictr.org.cn/edit.aspx?pid=141208&htm=4 (Identifier: ChiCTR2100053794, registered on December 3, 2021). If there are any modifications to the study design, the Ethics Committee will be notified promptly. The purpose and potential risks of this clinical trial will be fully explained to the participants’ relatives (or their legal representatives), from whom informed consent will be obtained.

We will disseminate the results of this study in meetings or publications when the trial is completed.

## 3 Discussion

TBE, the most frequent cause of death after TBI, is an independent risk factor for neurological unfavorable prognosis.^[3,4]^ TBE occurs commonly as pericontusional edema of brain contusions, which leads to a life-threatening increase in ICP. In evidence-based medicine, no treatment measures other than craniotomy and mannitol have been recommended for TBE.^[6]^ Thus, more effective and safe strategies to improve the outcome of patients with TBE are urgently needed. As a complementary and alternative therapy, acupuncture has been widely used to treat neurological diseases, and the effect of acupuncture on patients with TBI has been certified by some studies.^[28,29]^

Here, we describe the protocol of a single-center, single-blinded, RCT to determine if the addition of complementary acupuncture for TBE on acute phase is more effective than conventional treatment alone. We also aim to determine whether acupuncture has an anti-inflammatory effect and is associated with a reduction in the volume of brain edema. This RCT selects appropriate participants and controls confounding factors to optimize clinical evaluation of efficacy.

In this RCT of conventional treatments combined with acupuncture for TBE, we chose drug therapy (i.e., mannitol) as the conventional treatment to reduce ICP, because mannitol is commonly used clinically and is recommended by evidence-based medicine. Also, the optimal timing of acupuncture therapy for TBE is unclear. We relied on expert consensus and clinical guidelines that considered the safe and feasible approach when acupuncture therapy started within 72 hours after injury.^[30,31]^

In this study, the acupuncture points were selected based on traditional Chinese medicine theory, our previous pilot study, and summaries of the medical experts’ experiences. For example, the acupuncture points of GV26, PC6, and SP6 were selected on the basis of Shi's Xingnaokaiqiao theory, which has achieved significant effects in the treatment of acute and critical cerebrovascular diseases.^[32]^ In addition, Chen has used the acupuncture points of GV20, GV26, GB20, LI4, and LR3 to treat neurological diseases and achieved satisfactory clinical curative effects in improving brain function.^[33]^

Among the scores measuring functional recovery and quality of life after TBI, GOS is the most reliable, most validated, and most used score in RCTs.^[34]^ We defined the GOS at 6 and 12 months as the primary outcome.

The treatment plans for patients with TBE and the measurement of treatment effect are usually based on evaluating the ICP level at an acute-phase TBI. The ICP measure is commonly estimated by clinical judgment according to neurofunctional evaluation and CT image interpretation.^[18,35]^

This feasibility trial has the necessary optimizing features that may be helpful for future trial designs in TBE. However, some limitations still exist in this study. First, the number of cases is relatively small and is collected from a single center, which may have led to overfitting of the protocol. Second, we are adopting a single-blinding approach; because of the characteristics of acupuncture, the therapists and the participants cannot be blinded in this study. Finally, limiting participation of patients of age 16 to 75 years and with GCS scores of 6 to 12 means that the study results cannot be applied to all patients with TBI.

In conclusion, this pragmatic study protocol will allow us to determine the actual effects of acupuncture during real-life conditions in normal medical practice for TBI. Positive results from our study may lead to insights about a valuable complementary acupuncture option for TBE and could provide pilot evidence for additional and larger RCTs. This study has the potential to change recommendations for acute-phase acupuncture as a treatment for patients with moderate to severe TBI.

## Acknowledgment

We thank patients and relatives, physicians, nursing staff, and clinical research associates of the participating in this study.

## Author contributions

All authors approved the final version of the manuscript.

**Conceptualization:** Yong Huang.

**Formal analysis:** Zi-Quan Guo, Hong-Mei Gu.

**Project administration:** Wen-Bin Wang, Tai-Dong Chen.

**Writing – original draft:** Zi-Quan Guo, Hua Jiang.

**Writing – review & editing:** Yong Huang, Hong-Mei Gu.
